# Components of the Hematopoietic Compartments in Tumor Stroma and Tumor-Bearing Mice

**DOI:** 10.1371/journal.pone.0018054

**Published:** 2011-03-25

**Authors:** HoangDinh Huynh, Junke Zheng, Masato Umikawa, Robert Silvany, Xian-Jin Xie, Catherine J. Wu, Martin Holzenberger, Qianming Wang, Cheng Cheng Zhang

**Affiliations:** 1 Departments of Physiology and Developmental Biology, University of Texas Southwestern Medical Center, Dallas, Texas, United States of America; 2 Department of Clinical Sciences, University of Texas Southwestern Medical Center, Dallas, Texas, United States of America; 3 Dana-Farber Cancer Institute, Boston, Massachusetts, United States of America; 4 INSERM U515, Hopital St-Antoine, Paris, France; 5 School of Chemistry and Environment, South China Normal University, Guangzhou, People's Republic of China; The University of Hong Kong, Hong Kong

## Abstract

Solid tumors are composed of cancerous cells and non-cancerous stroma. A better understanding of the tumor stroma could lead to new therapeutic applications. However, the exact compositions and functions of the tumor stroma are still largely unknown. Here, using a Lewis lung carcinoma implantation mouse model, we examined the hematopoietic compartments in tumor stroma and tumor-bearing mice. Different lineages of differentiated hematopoietic cells existed in tumor stroma with the percentage of myeloid cells increasing and the percentage of lymphoid and erythroid cells decreasing over time. Using bone marrow reconstitution analysis, we showed that the tumor stroma also contained functional hematopoietic stem cells. All hematopoietic cells in the tumor stroma originated from bone marrow. In the bone marrow and peripheral blood of tumor-bearing mice, myeloid populations increased and lymphoid and erythroid populations decreased and numbers of hematopoietic stem cells markedly increased with time. To investigate the function of hematopoietic cells in tumor stroma, we co-implanted various types of hematopoietic cells with cancer cells. We found that total hematopoietic cells in the tumor stroma promoted tumor development. Furthermore, the growth of the primary implanted Lewis lung carcinomas and their metastasis were significantly decreased in mice reconstituted with IGF type I receptor-deficient hematopoietic stem cells, indicating that IGF signaling in the hematopoietic tumor stroma supports tumor outgrowth. These results reveal that hematopoietic cells in the tumor stroma regulate tumor development and that tumor progression significantly alters the host hematopoietic compartment.

## Introduction

Solid tumors are composed of cancerous and non-cancerous cells. The non-cancerous cells, including endothelial cells, hematopoietic cells, fibroblasts, myofibroblasts, pericytes, and mesenchymal stem cells, collectively form the cancer stroma or microenvironment [1], [2]. These stromal cells come from the local environment or from bone marrow (BM) via the circulation system and appear to provide important support for cancer cell growth and metastasis [1], [2]. For instance, BM-derived cells are recruited to the cancer site to stimulate outgrowth of tumors and form angiogenic and pre-metastatic niches for cancer growth [3], [4], [5]. Stromal fibroblasts and mesenchymal stem cells also play critical roles in angiogenesis and metastasis, respectively [6], [7]. However, the exact compositions and functions of the microenvironment that surround solid cancer are still largely unknown. Since a tumor cannot develop without the parallel expansion of a tumor stroma, the lack of understanding of this cancer microenvironment has severely hampered cancer research and the development of effective therapeutic approaches.

There is ample evidence that certain differentiated hematopoietic cells, including macrophages, T cells, and mast cells, are incorporated into the tumor microenvironment [1], [2]; however, a systematic investigation of the composition of the hematopoietic compartment of the tumor stroma has not been carried out. Hematopoiesis in vertebrates is a hierarchically organized developmental process in that highly specialized differentiated cells, including progenitors, precursors, and different lineages of blood cells, originate through an ordinate maturation program from the primitive hematopoietic stem cells (HSCs) [8]. Of these cells, HSCs are defined by their ability to self-renew and to differentiate into all blood cell types, whereas various progenitors posses much more limited self-renewal capacity and differentiation potential. In adults, HSCs mainly reside in BM; a small fraction also circulate in the blood stream and can be found in extramedullary organs including spleen and liver [8], [9]. The flow cytometry-based surface phenotype analysis and various functional assays, including the BM reconstitution analysis, remain the assays of choice for the analysis of the presence and activities of various hematopoietic cell types [8].

We sought to determine the composition and function of hematopoietic cells in tumor stroma and to determine whether tumor development affects the hematopoietic compartment of a tumor-bearing host. These studies are of fundamental importance to our understanding of the basic molecular and cellular mechanisms of tumor pathogenesis. A more complete understanding of the tumor microenvironment will make possible novel types of anti-tumor therapy.

## Results

### Various hematopoietic populations exist in tumor stroma

**Figure 1:**
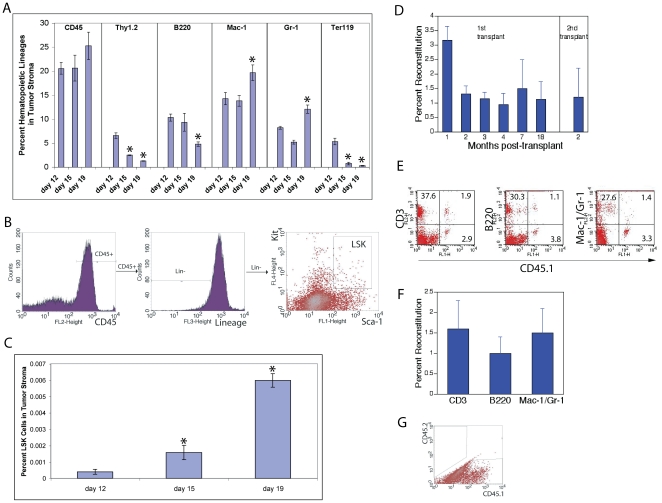
Analysis of the hematopoietic compartment of LL2 tumor stroma. Fig. 1A–C show the results of flow cytometry staining for hematopoietic cell surface antigens in dissociated tumor masses arising after the subcutaneous injection of 10^6^ LL2 cells into C57BL/6 CD45.1 host mice at indicated days post-implantation. (A) Flow cytometry analysis of hematopoietic cells and their major lineages in LL2 tumor stroma at day 12, 15, and 19 after implantation as total hematopoietic cells (CD45), T cells (Thy1.2), B cells (B220), myeloid cells (Mac-1 and Gr-1), and erythroid cells (Ter119^+^) (n = 5). * significantly different from day 12 values. (B) Representative flow cytometry plots showing that CD45^+^Lin^−^Sca-1^+^Kit^+^ (LSK) cells exist in LL2 tumor stroma. (C) Frequencies of CD45^+^Lin^−^Sca-1^+^Kit^+^ cells in LL2 tumor stroma at day 12, 15, and 19 after implantation (n = 5). * significantly different from day 12 values. (D) HSCs in the LL2 tumor stroma have long-term repopulation ability and repopulate secondary recipients. Lethally irradiated CD45.2 congenic mice were injected with 1×10^5^ CD45.2 bone marrow competitor cells and 1×10^6^ CD45.1 cells, isolated from the LL2 tumor mass. Shown are the repopulation activities of hematopoietic populations derived from the cancer stroma in long-term reconstitution (n = 6) and secondary reconstitution experiments (n = 5). (E–F) Donor repopulation in T lineage (CD3), B lineage (B220), and myeloid lineage (Mac-1/Gr-1) in peripheral blood in the experiment described in panel D at 7 months post-transplant (n = 6). (G) Hematopoietic cells in tumor stroma originate from host BM. 1,000,000 CD45.1 donor BM cells were transplanted into lethally irradiated CD45.2 C57BL/6 mice. At 4 months post-transplant, 10^5^ LL2 tumor cells were subcutaneously implanted into these mice. Three weeks later, flow cytometry was used to characterize the hematopoietic cells in tumor stroma. A representative flow cytometry plot shows 100% of the tumor stromal CD45^+^ cells were CD45.1^+^.

Using the Lewis lung carcinoma (LL2) implantation mouse model, we characterized the hematopoietic compartment in the tumor stroma. Figures 1A–C and Table 1 show the results of staining for hematopoietic cell surface antigens in dissociated tumor masses arising after the subcutaneous injection of 10^6^ LL2 cells into C57BL/6 CD45.1 host mice at various time points post-implantation. The composition of hematopoietic cells in tumor stroma differed from that from host peripheral blood (PB) or BM (Table 1), indicating that there exists a unique hematopoietic compartment in tumor stroma. The percentage of hematopoietic cells in the LL2 tumor stroma modestly increased from 20.6% at day 12 to 25.3% at day 19 after tumor implantation (Fig. 1A). The relative composition of myeloid cells (Mac-1^+^ and Gr-1^+^) increased whereas that of lymphoid cells (T cells as Thy1.2^+^ and B cells as B220^+^) and erythroid cells (Ter119^+^) in this compartment decreased over time (Fig. 1A).

**Table 1:** Repopulating hematopoietic stem cells and differentiated hematopoietic cells exist in LL2 tumor stroma.

1	2	4	5	6	7	8	9	10
	CD45^+^ %	Thy1.2^+^ %	B220^+^ %	Mac-1^+^ %	Gr-1^+^ %	Ter119^+^ %	CFU-GM (per 1 million cells)	CD45^+^Lin^−^Sca-1^+^Kit^+^ %
Tumor stroma	25.3±2.8	1.3±0.1	4.8±0.4	19.7±1.6	12.1±0.9	0.3±0.1	3.5±0.5	0.006±0.001
Tumor peripheral blood	98.7±2.4	11.7±1.9	13.1±2.2	74.5±3.7	66.4±4.5	15.0±5.6	N/D	0.009±0.002
Tumor bone marrow	77.0±2.0	0.9±0.2	5.5±2.5	70.5±3.8	61.1±4.7	19.4±4.2	2962±82	0.2±0.02
Normal peripheral blood	99.3±2.2	30.7±9.7	39.5±2.7	26.5±12.2	19.3±10.7	28.9±3.6	N/D	0.0009±0.0005
Normal bone marrow	69.7±2.9	2.8±0.2	20.7±1.3	44.0±4.4	41.5±4.9	32.5±2.5	1425±32	0.08±0.05
Normal muscle	1.6±0.2	0.8±0.1	0.1±0.01	0.2±0.01	0.5±0.04	0.1±0.01	0±0	0.0±0.0

Data from tumor mice were obtained from dissociated tumor masses arising after the subcutaneous injection of 10^6^ LL2 cells into C57BL/6 CD45.1 host mice at day 19 post-implantation (n = 5).

We also measured the existence of hematopoietic progenitors and phenotypic HSCs in the tumor stroma. The number of progenitors for granulocytes and monocytes (CFU-GM) was low, with 3.5 per 1,000,000 tumor cells at day 19 post-implantation (Table 1). Interestingly, in the LL2 tumor stroma, we found phenotypic HSCs, measured as CD45^+^Lin^−^Sca-1^+^Kit^+^ cells (Fig. 1B). The flow cytometry analyses indicate that there were few Lin^−^Kit^+^ cells in the tumor stroma, concordant with a previous report [3]. The average frequency of these phenotypic HSCs increased over time from 0.0004% at day 12, to 0.0016% at day 15, and to 0.006% at day 19 (Fig. 1C). In particular, the frequency of phenotypic HSCs detected in the tumor stroma (0.006±0.001%) at day 19 was 1/13 or 1/33 of that in BM of normal mice or tumor-bearing mice respectively (0.08±0.05 and 0.20±0.02% respectively) (Table 1). Since the tumors were perfused, and the cell composition including the percentage of HSCs in tumor stroma was quite different from that in peripheral blood, our result indicates that phenotypic HSCs reside in tumor stroma and are not contaminant from blood.

Since the surface phenotype of HSCs in extramedullary organs can be different from that of BM HSCs [9], we used BM reconstitution analysis, the “gold standard” for measuring HSC repopulating activity, to determine whether functional HSCs existed in the tumor stroma. As we performed before [10], [11], [12], [13], [14], [15], [16], we examined the HSC activity of the donor cells in competitive reconstitution analyses, in which lethally irradiated recipient mice were co-transplanted with both the donor cells to be tested and WT BM competitors. The competitor cells serve as an internal control and as a supply of hematopoietic cells until the transplanted stem cells can generate sufficient mature lymphoid and myeloid cells for survival. Donor and recipient mice are genetically identical except for the CD45 surface protein that is found on nucleated peripheral blood cells and that is not involved in hematopoiesis or stem cell activity; donor cells carried the marker CD45.1, while recipient mice and supportive cells expressed CD45.2. An extremely stringent 18-month competitive reconstitution analysis and a secondary transplantation showed that the tumor stroma contained long-term repopulating HSCs (Fig. 1D). These cells were capable of repopulating both lymphoid and myeloid lineages in long-term reconstitution analysis (Fig. 1E–F), attesting that these are functional HSCs. The relative low repopulation of the donor cells in all lineages compared to BM competitors suggests these HSCs in the tumor stroma have weak engraftment to the recipient BM. Nevertheless, our data, for the first time to our knowledge, demonstrated that functional HSCs exist in tumor stroma.

To determine whether the hematopoietic cells in tumor stroma originated from the BM or from the local environment, we transplanted CD45.1 C57BL/6 donor BM cells into lethally irradiated CD45.2 C57BL/6 mice. At 4 months post-transplant, the recipient BM was completely repopulated by the donor CD45.1 cells, whereas peripheral tissues contained mostly CD45.1 cells with certain CD45.2 cells as reported [9]. We implanted LL2 tumor cells subcutaneously into these mice. Three weeks later, we isolated tumors and used flow cytometry to characterize the hematopoietic cells in stroma. We found that all of the tumor stromal CD45^+^ cells were CD45.1^+^ (Fig. 1G). Accordant with previous studies [3], we concluded that hematopoietic cells in the LL2 stroma are derived from BM.

### HSC numbers dramatically increase in tumor-bearing mice

**Figure 2:**
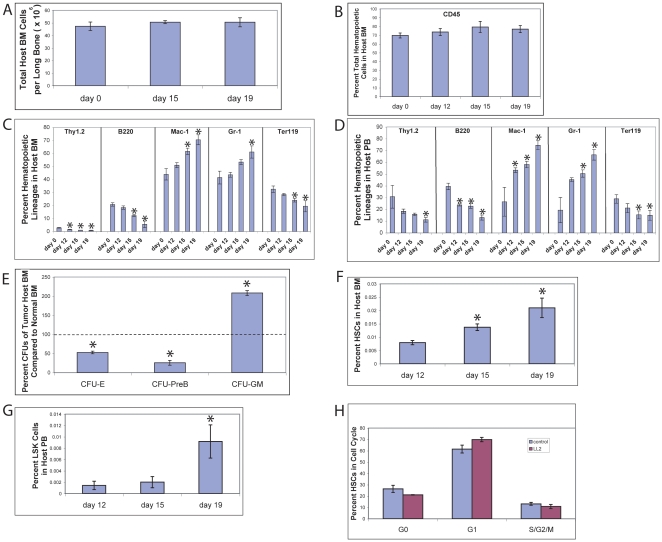
Analysis of the hematopoietic compartment of the LL2 tumor-bearing mice. (A–G) Total BM cells (A), total hematopoietic cells in BM (B), hematopoietic lineages in BM (C), hematopoietic lineages in PB (D), hematopoietic progenitors in BM (E), CD45^+^Lin^−^Sca-1^+^Kit^+^CD34^−^FLK2^−^ cells in BM (F), and CD45^+^Lin^−^Sca-1^+^Kit^+^cells in PB (G) at indicated days in C57BL/6 CD45.1 host mice before and after the subcutaneous injection of 10^5^ LL2 cells were analyzed by flow cytometry or colony assays (n = 5). * significantly different from day 0 or normal values (for panel B–E), or from day 12 values (for panel F–G). (H) The cell cycle status of BM HSCs in tumor-bearing mice at day 19 post-implantation does not significantly differ from that of counterparts in normal mice. HSCs as Lin^−^Sca-1^+^Kit^+^Flk2^−^CD34^−^ cells were stained with Hoechst 33342 and pyronin Y, and analyzed for cell cycle stage (n = 5).

Next we sought to determine whether the host hematopoietic compartment was affected by tumor growth. Total cellularity and total hemtopoietic cell counts of tumor-bearing mice did not significantly change over time (Fig. 2A–B). However, in both BM and PB of tumor-bearing mice we observed significantly increased percentages of myeloid (Mac-1^+^ and Gr-1^+^) cells but decreased lymphoid (Thy1.2^+^ and B220^+^) and erythroid (Ter119^+^) cells as a function of time post-implantation (Fig. 2C–D). This trend is similar to what occurred in the hematopoietic lineages in tumor stroma. In addition, we analyzed the frequencies and numbers of hematopoietic progenitors and phenotypic HSCs in the tumor-bearing mice and healthy controls. CFU-GM increased approximately 2-fold whereas CFU-E and CFU-Pre-B decreased at least 50% in tumor-bearing mice compared to normal mice (Fig. 2E), concordant with the total increase of myeloid cells and decrease of lymphoid and erythroid cells. The BM and PB enriched phenotypic HSCs as Lin^−^Sca-1^+^Kit^+^CD34^−^Flk-2^−^ or Lin^−^Sca-1^+^Kit^+^ cells increased over time. Numbers of BM HSCs increased about 3-fold and PB HSCs increased 7-fold at day 19 relative to numbers on day 12 (Fig. 2F–G). This suggests that the presence of a tumor induces *in vivo* expansion of HSCs and progenitors in the BM, which leads to increased HSCs and progenitors in the circulation. Consistent with this result, the tumor-bearing mice display splenomegaly, with 2-fold increase of spleen size and weight compared to healthy controls (data not shown).

Because numbers of HSCs in the tumor-bearing mice significantly increased, we tested whether the cell cycle of HSCs in the host BM changed. As shown in Figure 2H, there was no significant changes in fractions of cells in given cell cycle stages in HSCs in the BM of tumor-bearing mice at day 19 compared to normal mice, suggesting that the tumor did not significantly alter the quiescence of host BM HSCs at the late stage of cancer development.

### Co-implanted hematopoietic cells from tumor stroma promote tumor development

**Figure 3:**
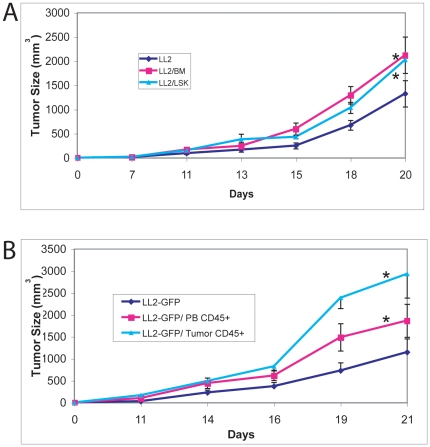
Tumor stromal hematopoietic cells stimulate LL2 tumor growth. (A) Co-implanted HSCs or total BM cells stimulate the growth of primary LL2 tumor. 100,000 GFP-marked LL2 cells were co-implanted with with 1×10^4^ CD45.1 total BM cells or enriched normal bone marrow HSCs (as Lin^−^Sca-1^+^Kit^+^ cells) subcutaneously into C57BL/6 CD45.2 host mice (n = 8). During the 3-week period of analysis, the size of the primary tumor was measured. (B) Tumor stromal hematopoietic cells stimulate tumor growth. 100,000 tumor stromal CD45^+^ cells or PB cells from tumor-bearing mice were collected by FACS and co-implant with 10^5^ LL2 cells into healthy mice (n = 5). Shown are the sizes of the primary tumors. Tumor growth curves for different experimental groups were compared using the Generalized Estimating Equations (GEE) method with AR(1) correlation structure. * significantly different from LL2-GFP growth curve, ** significantly different from LL2-GFP and LL2/PB CD45^+^ growth curves, p<0.05.

We developed an assay to compare the abilities of different hematopoietic populations to collaborate functionally with tumor cells to affect the tumor development. In this assay, we use FACS to isolate a certain population of hematopoietic cells and mix them with a fixed number of LL2 cancer cells prior to implantation into the C57BL/6 mice. The kinetics of tumor growth was determined to evaluate the tumor-promoting ability of the co-implanted hematopoietic cells. In the experiment summarized in Figure 3A, we co-implanted 1×10^5^ LL2 cells with 1×10^4^ CD45.1 total BM cells or with enriched normal BM HSCs as Lin^−^Sca-1^+^Kit^+^ cells subcutaneously into C57BL/5 CD45.2 host mice. During the 3-week period of analysis, the tumor size was measured. These data show that both total BM cells and the enriched HSC population positively regulated tumor growth. It is noteworthy that the co-implantation of total BM cells with LL2 cells led to similar level of tumor size as the same number of transplanted LSK cells. Clearly, differentiated hematopoietic cells did promote tumor progression. This suggests that additional HSCs in the tumor local environment do not necessarily further promote tumor growth.

We further determined the effect of hematopoietic cells isolated from the tumor stroma on cancer development. Because it was technically difficult to isolate the low frequent Lin^−^Sca-1^+^Kit^+^ cells from these tumors, we isolated hematopoietic cells from LL2 tumor stroma or host PB as a control and co-injected them with GFP^+^LL2 tumor cells into secondary mice. We found that, although the host PB CD45^+^ cells stimulated LL2 tumor growth compared to LL2 cells alone, CD45^+^ cells isolated from previously existing tumor stroma had significantly increased ability to enhance LL2 tumor growth than these PB CD45^+^ cells (Fig. 3B, *p*<0.05). This result suggests that 1) hematopoietic cells in tumor stroma enhance tumor growth, 2) hematopoietic cells from tumor stroma are different from those from PB in promoting tumor growth, not unexpectedly as the compositions of these two sources of cells are different (Table 1), and 3) tumor stroma has certain “educating” effect on hematopoietic cells that leads to tumor development.

### IGF-IR expressed on cells from hematopoietic stroma is important for tumor development and metastasis

To further validate the pathological effect of the hematopoietic cells in the tumor stroma, we studied the role of IGF signaling in these hematopoietic compartment. We previously showed that the the receptor for IGF-2 is expressed on the surface of all HSCs [10]. IGF type I receptor (IGF-IR) is the signaling receptor for insulin-like growth factor 1 and 2 (IGF-1 and IGF-2). The IGF pathway has been reported to play important roles in the development of a range of malignancies, including both non-small cell lung cancer and small cell lung cancer (see review [17]). For example, elevated plasma levels of IGF-1 and single nucleotide polymorphisms within the IGF axis are associated with an increased risk of lung cancer [17]. The activation of IGF-IR facilitates malignant transformation and the majority of IGF-2 transgenic mice develop lung cancer by 18 months of age [17]. Of note is that these studies were focused on the activity of IGF-IR that is expressed on cancer cells. We sought to test whether IGF-IR expressed by the hematopoietic stroma plays any role in the development of the LL2 tumors.

**Figure 4:**
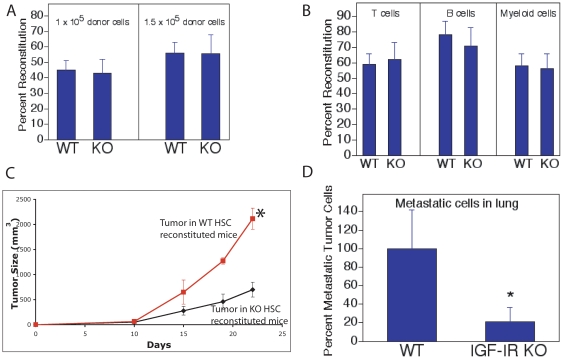
Mice with the IGF-IR^−/−^ tumor stromal HSCs had slower tumor development in the LL2 implantation model than mice reconstituted with wild-type HSCs. (A) C57BL/6 CD45.2 E15.5 IGF-IR^−/−^ fetal liver HSCs had similar engraftment as wild-type HSCs in CD45.1 recipient mice in a competitive repopulation analysis at 4 months post-transplant (n = 7). CD45.1 total BM cells were used as competitors. (B) IGF-IR^−/−^ HSCs had similar differentiation into T, B, and myeloid lineages as wild-type HSCs at 4 month post-transplant (n = 7). (C) Mice with the IGF-IR^−/−^ tumor stromal HSCs had slower tumor growth in the LL2 implantation model. Shown is a representative result of three independent experiments of tumor growth measured after 1×10^6^ LL2 cells were implanted subcutaneously into IGF-IR^−/−^ or wild-type HSCs reconstituted mice at 4 month post-transplant (n = 5). * significantly different from the KO growth curve, p<0.05. (D) Mice with the IGF-IR^−/−^ tumor stromal HSCs had dramatically decreased GFP^+^ LL2 tumor cell metastasis in lung compared to those reconstituted with wild-type HSCs. Results shown were from pooled data from three experiments (in each experiment n = 3–5). Tumor growth curves for different experimental groups were compared using the Generalized Estimating Equations (GEE) method with AR(1) correlation structure. * significantly different from wild-type value, p<0.05.

Although IGF-IR^−/−^ mice die after birth [18], [19], we were able to collect IGF-IR^−/−^ HSCs from the fetal liver. Then we reconstituted recipient mice with IGF-IR^−/−^ or wild-type fetal liver HSCs. Four months later, the recipients were fully repopulated by the donor IGF-IR^−/−^ or wild-type HSCs, and we implanted LL2 cancer cells into these mice. The IGF-IR^−/−^ HSCs had no apparent defect in engrafting the recipient mice (Fig. 4A), nor did they have a noticeable skew in lineage differentiation compared to HSCs from wild-type mice (Fig. 4B). Nevertheless, LL2 tumors grew significantly more slowly in these IGF-IR^−/−^ HSC reconstituted mice than in mice engrafted with wild-type HSCs (Fig. 4C, *p*<0.05), accompanying with dramatically decreased metastasis to the lung (Fig. 4D). This experiment suggests that the lack of IGF signaling in the hematopoietic compartment of tumor stroma hampers the solid tumor development. This novel result complements the conventional view that IGF signaling in tumor cells *per se* is important for cancer development.

## Discussion

Since cancer pathogenesis involves a concerted interplay between the tumor and the microenvironment, it is desirable to elucidate the roles of tumor stroma in tumor development. In this study, we sought to determine the composition and potential function of hematopoietic cells in the stroma of solid tumors and in tumor-bearing mice. To this end, we used an LL2 implantation tumor model. Cells of the LL2 line are advantageous because they produce tumors in syngeneic C57BL/6 mice, which are ideal for quantitating HSCs by reconstitution analysis based on congenic CD45.1 and CD45.2 markers. Because LL2 cells are syngeneic with their hosts, their tumorigenicity can proceed in the presence of a fully competent host immune system. This is especially important in a study of the hematopoietic compartment of the tumor stroma and the tumor-bearing host. Moreover, LL2 cells, when injected subcutaneously, can form lung metastases. This makes it possible to compare the effects of different stromal components on regulation of potential tumor cell migration.

Here we provide evidence that functional hematopoietic progenitors and HSCs exist in tumor stroma. Although the very low frequency of HSCs in tumor stroma makes them impossible to observe by immunohistochemistry, we were able to detect these cells using flow cytometry analysis and the “gold standard” BM reconstitution assay. To our knowledge, this was the first demonstration that functional HSCs exist in tumor stroma. It is apparent that these tumor stromal HSCs are not contaminants from PB: this was assured by our approach to isolate hematopoietic cells from perfused tumors and was also attested by the demonstration of different frequencies of various hematopoietic populations in tumor stroma and PB. Nevertheless, these tumor stromal hematopoietic cells do originate from BM and may be recruited to tumor sites from PB through inflammatory signals. Our result is supported by the emerging evidence showing that HSCs or progenitors can themselves home to sites of inflammation to rapidly produce cells that are essential for the immune response [20]. Concordant with the idea that tumors release certain endocrine signals that change the representation of stem cells or progenitors in other tissues or organs [3], we showed that the tumor-bearing mice had 3-fold and 7-fold increases in of BM and PB HSCs, respectively. Similarly, during tumor development, the percentage of faster proliferating myeloid cells (including myeloid progenitors) increased and that of slower growing lymphoid cells decreased over time in host BM and PB. In summary, the whole process appears to be as follows. Tumor-produced hormone signals reach BM, thus BM HSCs increase. The increased proliferation of BM HSCs exceeds the capacity of HSC microenvironment leading to HSC mobilization and increases in numbers of PB HSCs. These BM-derived hematopoietic cells including HSCs are eventually recruited to tumor and become the source of hematopoietic cells in tumor stroma. A better understanding of the alteration of the hematopoietic compartment in host and tumor stroma during tumor progression may lead to new strategies for cancer treatment. For instance, the effective retention of BM HSCs in their BM niche or block of the migration of BM-derived hematopoietic cells in cancer patients should negatively control the cancer development.

Is the existence of non-cancerous cells in tumor stroma a consequence or a cause of tumor development? So far numerous lines of prior evidence already indicate that stromal cells play important roles in tumor progression. Endothelial cells recruited to the tumor mass support neovascularization. BM-derived cells have been shown to support tumor outgrowth and form pre-metastatic niches [3], [4], [5]. Stromal fibroblasts and mesenchymal stem cells also support in angiogenesis and metastasis, respectively [6], [7]. Our studies provide further evidence that tumor stromal hematopoietic cells regulate the tumor growth and metastasis. We found that the co-implantation of normal HSCs with cancer cells in this tumor model promoted tumor growth. Since total BM cells had similar ability to stimulate co-implanted tumor growth as LSK cells, HSCs may not directly affect tumor growth; instead, it may be hematopoietic cells that differentiate in the tumor microenvironment that play a critical role in tumor outgrowth. Nevertheless, it seems that a combination of multiple lineages of hematopoietic cells instead of a certain single lineage is advantageous to promoting tumor development. When we co-implanted individual lineage cells such as CD3^+^ (T cells), B220^+^ (B cells), Mac-1^+^ (monocytes), Gr-1^+^ (granulocytes), or Ter119^+^ cells (red blood cells) with LL2 cells, we found their tumor-promoting effects were not as potent as LSK cells or total BM cells (data not shown). This suggests that the full lineage spectrum that is derived from HSCs locally may have the strongest activity in tumor promotion. Consistent with this view, we showed that previously “educated” tumor stromal total hematopoietic cells had greater ability to support tumor growth than circulating hematopoietic cells.

The IGF ligands IGF-1 and IGF-2 bind to their common signaling receptor IGF-IR and initiate a variety of signaling events. It is known that IGF-IR regulates cell growth, survival, adhesion, and motility [17]. Our previous work demonstrated that all normal HSCs express the receptor for IGF-2 and that IGF-2 stimulates *ex vivo* expansion of these normal HSCs [10]. In the present study, we found that tumor stromal HSCs are originally derived from BM. Although IGF-IR^−/−^ HSCs do not appear to have overt defects in hematopoietic engraftment and differentiation, the incorporation of IGF-IR^−/−^ hematopoietic cells into the tumor stroma significantly hampered tumor growth and metastasis. This suggests that IGF-IR may regulate certain cell fates (division, differentiation, migration, or apoptosis) or activities (such as immune response) of hematopoietic cells under inflammation stress in the unique tumor stromal environment. Overall our results reveal that, in addition to its direct role in cancer survival and growth, IGF signaling in tumor stroma is also important for solid cancer development. These results do not contradict but rather complement the conventional view that IGF signaling is important for cancer development.

Questions regarding the repopulating hematopoietic cells in tumor stroma still remain. For example, what is the mechanism for LL2 stroma to maintain the primitive status of a small fraction of HSCs? One possibility is that HSCs are supported by the growth factors, cytokines, chemokines, or membrane proteins produced by the tumor stromal environment. As we previously demonstrated, certain tumor cells can secrete proteins to support HSCs *ex vivo* [14]. It is therefore reasonable to assume that, while the LL2 stromal environment largely supports HSC differentiation *in vivo*, it possesses a certain capacity to maintain a very small number of primitive HSCs possibly for its own sake. A systematic study of the effects of the tumor stroma-produced factors on cell fates of HSCs will be necessary to find the answer. Another question is, what is the exact phenotype of HSCs in tumor stroma? Is it the same as that of BM HSCs? We need to emphasize that, although we identified the existence of CD45^+^Lin^−^Sca-1^+^Kit^+^ cells (as the phenotype of enriched BM HSCs) in the LL2 stroma, we are uncertain if this represents the actual phenotype of these HSCs. That is why we used the “gold standard” reconstitution analysis to confirm the existence of functional repopulating HSCs in this study. Given the fact that HSCs change their surface phenotype in stressed conditions, culture, or extramedullary tissue such as in the liver [9], [11], future investigations coupling FACS-based cell fractionation with BM reconstitution analysis will be needed to clarify this issue. Other questions include, whether our observation of hematopoietic compartments in the LL2 tumor stroma can be applied to other tumor models, metastatic cancers, and even human cancers. Since one of the key features of hematopoietic cells is their ability to migrate and access to various tissues and organs, we hypothesize that this ability may contribute to the formation of clusters of hematopoietic cells that have been “educated” by the primary tumor to serve as metastatic microenvironment in distant locations. It thus will be interesting to study the relationship between hematopoietic cells in the primary cancer and the stroma of the metastatic cancer. It will also be important to determine the hematopoietic compositions of human tumor stroma.

## Materials and Methods

### Ethics Statement

All animal experiments were performed with the approval of UT Southwestern Committee on Animal Care (APN# 2007-0068).

### Cell lines, animals, and tumor implantation and measurement

Murine Lewis lung carcinoma (LL2) cells were obtained from the ATCC and cultured under standard conditions. Retroviral MSCV-GFP was introduced into LL2 cells to produce stable GFP^+^ LL2 cells. C57BL/6 CD45.2 and CD45.1 mice were purchased from the Jackson Laboratory or the National Cancer Institute. IGF-IR^−/+^ mice as previously described [19] were in pure C57BL/6 background. All animals were maintained at the University of Texas Southwestern Medical Center animal facility and animal experiments were performed with the approval of UT Southwestern Committee on Animal Care. Tumor cells were injected subcutaneously into mice and mice were maintained for about 3 weeks. Tumor size was measured on the flanks of live mice using calipers; volume was calculated as (length of tumor)×(width of tumor)^2^/2. To analyze lung metastasis, entire lungs were harvested and single cell suspensions were prepared by collagenase treatment. GFP^+^ LL2 cells originating from the distant implanted tumor were counted by flow cytometry analysis. The flow cytometry result was confirmed by counting GFP^+^ surface foci of the harvested lung under a dissecting microscope.

### Preparation of hematopoietic cells from tumors

Mice were perfused with cold PBS and primary tumors were removed and chopped with a McIlwain Tissue Chopper (Mickle Laboratory Engineering Company). The tissue was washed with PBS and then placed in collagenase-dispase medium (Liver Digest Medium, Invitrogen) at 37°C for 90 min as we described previously [21]. Cells passed through a 70-µm strainer were used for further flow cytometry analysis or sorting.

### Flow cytometry

BM and PB cells were isolated from 5–8 week old C57BL/6 mice. Lin^−^Sca-1^+^Kit^+^ or Lin^−^Sca-1^+^Kit^+^CD34^−^Flk-2^−^ cells were isolated by staining with a biotinylated lineage cocktail (anti-CD3, anti-CD5, anti-B220, anti-Mac-1, anti-Gr-1, anti-Ter119, and anti-7-4; Stem Cell Technologies) followed by streptavidin-PE/Cy5.5, anti-Sca-1-FITC, and anti-Kit-APC, and anti-CD34-PE and anti-Flk-2-PE if necessary. To analyze hematopoietic lineages and repopulation of mouse HSCs, mouse peripheral blood cells were collected by retro-orbital bleeding, followed by lysis of red blood cells and staining with anti-CD45.2-FITC, and anti-CD45.1-PE, and anti-Thy1.2-PE (for T-lymphoid lineage), anti-B220-PE (for B-lymphoid lineage), anti-Mac-1-PE, anti-Gr-1-PE (cells co-staining with anti-Mac-1 and anti-Gr-1 were deemed to be of the myeloid lineage), or anti-Ter119-PE (for erythroid lineage) monoclonal antibodies. All antibodies were from BD Pharmingen. The “percent reconstitution” shown in figures was based on the staining results of anti-CD45.2 and anti-CD45.1. In all cases flow cytometry analysis of hematopoietic lineages was also performed to confirm multilineage reconstitution as we described [14], [15], [16].

### Hematopoietic colony assays

CD45^+^ cells from LL2 tumor stroma or normal BM cells were resuspended in IMDM with 2% FBS and were then seeded into methylcellulose medium M3334 (StemCell Technologies) for CFU-E, M3434 (StemCell Technologies) for CFU-GM, or M3630 (StemCell Technologies) for CFU-Pre-B assays, according to the manufacturer's protocols and as described [16].

### HSC cell cycle analysis

The cell cycle analysis with Hoechst and pyronin Y staining was performed as described [16]. Briefly, the Lin^−^Sca-1^+^Kit^+^Flk2^−^CD34^−^ cells were collected in Hank's buffered salt solution medium containing 10% FBS, 1 g/liter glucose, and 20 mM Hepes (pH 7.2). Cells were washed, Hoechst 33342 (20 µg/ml, Invitrogen) was added, and cells were incubated at 37°C for 45 min after which pyronin Y (1 µg/ml, Sigma) was added. Cells were incubated for another 15 min at 37°C, washed, and resuspended in cold PBS. Samples were immediately analyzed by flow cytometry (BD Biosciences, FACSAria).

### Competitive reconstitution analysis

The indicated numbers of mouse CD45.2 or CD45.1 donor cells were mixed with 1 or 2×10^5^ freshly isolated CD45.1 or CD45.2 competitor BM cells, and the mixture were injected intravenously *via* the retro-orbital route into each of a group of 6–9 week old CD45.1 or CD45.2 mice previously irradiated with a total dose of 10 Gy. For secondary transplantation, CD45.1^+^ cells were collected from primary recipients and 10^6^ cells were injected with 10^5^ CD45.2 competitors into lethally irradiated secondary recipient mice. To measure reconstitution of transplanted mice, peripheral blood was collected at the indicated times post-transplant and the presence of CD45.1^+^ and CD45.2^+^ cells in lymphoid and myeloid compartments were measured [14], [16].

### Statistical analysis

Data are expressed as mean ± SEM. Tumor growth curves for different experimental groups were compared using the Generalized Estimating Equations (GEE) method with AR(1) correlation structure. Tumor sizes among different experimental groups were also compared at each time points using t-test. SAS 9.1.3 was used for the analysis. Data were considered statistically significant if *p*<0.05.
